# Environmental Lead (Pb) Exposure Versus Fatty Acid Content in Blood and Milk of the Mother and in the Blood of Newborn Children

**DOI:** 10.1007/s12011-015-0482-5

**Published:** 2015-08-28

**Authors:** Irena Baranowska-Bosiacka, Ida Kosińska, Dominika Jamioł, Izabela Gutowska, Adam Prokopowicz, Ewa Rębacz-Maron, Marta Goschorska, Tomasz Olszowski, Dariusz Chlubek

**Affiliations:** Department of Biochemistry and Medical Chemistry, Pomeranian Medical University, Powstańców Wlkp. 72 Str., Szczecin, Poland; Department of Biochemistry and Human Nutrition, Pomeranian Medical University, Broniewskiego 24 Str., Szczecin, Poland; Institute of Occupational Medicine and Environmental Health, Kościelna 13 Str., 41-200 Sosnowiec, Poland; Department of Vertebrate Zoology and Anthropology, University of Szczecin, Wąska 13 Str., 71-415 Szczecin, Poland; Department Hygiene and Epidemiology, Pomeranian Medical University, Powstańców Wlkp. 72 Str., Szczecin, Poland

**Keywords:** Pb, Fatty acid, Newborn

## Abstract

Significant progress in understanding the effects of the neurotoxic action of lead (Pb) in young organisms had led to reduction of “safe” level in the blood (Pb-B) to 5 μg/dL in children and pregnant women. Prolonged exposure to relatively low levels of Pb, generally asymptomatic and subclinical (i.e., microintoxication), is currently the dominant form of environmental poisoning, and its negative effects on health may appear after many years, e.g., secondary contamination from Pb bone deposits released in pregnancy. Therefore, the aim of this study was to investigate the effect of environmental exposure (urban areas) of mothers to Pb, on its levels in their milk and blood and in the blood of newborns. Moreover, the aim was to determine the fatty acid profile in the mothers’ blood and milk and in the blood of newborns. We also wanted to find if infant birth weight depends on Pb blood levels, as well as on Pb and fatty acid levels in the blood and milk of the mothers. Finally, we examined if the mothers’ weight and body mass index (BMI) before pregnancy influenced the concentration of Pb and fatty acid profile in the blood and milk of mothers and in the blood of their children. Analysis of fatty acids elaidic (C18:1, 9t), oleic (C18:1, 9c), vaccenic (C18:1, 11t), *cis*-vaccenic (C18:1, 11c), linoleic (C18:2, *cis*), γ-linolenic (C18:3, n-6), α-linolenic (C18:3, n-3), arachidonic (C20:4, n-6), eicosapentaenoic (C20:5, n-3), and docosahexaenoic (C22:6, n-3) was conducted by gas chromatography. The concentration of Pb in the whole blood and milk were determined by atomic absorption spectrometry with graphite furnace atomization and Zeeman correction. Our study established a significant and strong correlation between the content of Pb in the blood of the mother and the child. This supports the assumption that the transport of Pb through the placenta is neither regulated nor selective. Environmental maternal exposure to lead resulting in Pb-B levels considered safe for pregnant women had no effect on infant birth weight, the concentration of fatty acids in the blood and milk of mothers, or in the blood of newborns. Mothers’ weight and BMI before pregnancy had no effect on the concentration of Pb and studied fatty acid profile.

## Introduction

Lead (Pb) is a metal which is commonly present in the environment and exerts a toxic effect on many organs of the human body [1–3]. The concentration of Pb in living organisms is closely related to anthropogenic environmental contamination. In developed countries, growing awareness of its effects on human health has resulted in efforts to restrict its use [3, 4]. However, on a global scale, the total level of Pb emissions into the atmosphere is still high. Moreover, lead compounds are not biodegradable and therefore the current levels of environmental Pb contamination cannot be effectively reduced [4–6]. A primary target for lead is the developing central nervous system. Acute Pb contamination in children (lead concentration in whole blood—Pb-B >70 μg/dL), which is currently very rare, can have a dramatic effect on the central nervous system, i.e., brain edema, convulsions, coma, and lead encephalopathy [6]. Pb poisoning in children on a scale unheard of for decades has been detected in rural northwestern Nigeria. A total of 161 deaths in two villages have been attributed to an incident from May 2009 to May 2010, with hundreds and potentially thousands more people becoming seriously ill [7, 8].

Significant progress in understanding the effects of the neurotoxic action of Pb in young organisms had led to the creation of a system of diagnosis and treatment of lead poisoning and a gradual reduction of “safe” levels in the blood to 10 μg/dL [9, 10]. In children and pregnant women, the permissible dose is 5 μg/dL [3]. At the same time, prolonged exposure to relatively low levels of lead, generally asymptomatic and subclinical (i.e., microintoxication), is currently the dominant form of environmental poisoning and its negative effects on health may appear after many years, e.g., secondary contamination from Pb bone deposits released in pregnancy [11]. This means that pregnant women should be monitored for Pb exposure which may result in miscarriage, premature birth, low birth weight, and neonatal malformations [12, 13]. Many studies have also indicated that Pb levels considered safe can have a neurotoxic effect on the developing brain in the pre- and neonatal period of life [14, 15].

A woman’s body during pregnancy is burdened with additional functions. It must create the right environment and provide the necessary elements to the developing fetus. One such component is lipids; the lipid profile of a pregnant woman changes to provide energy security for both the mother and child [16]. Of particular importance are the fatty acids that play an important role as building blocks and a source of energy, acting also as a very important component for the proper development of the brain. After birth, the mother’s body plays an important role in securing energy through the production of milk with high amounts of fatty acids. Thus, the mother’s body during pregnancy and breast-feeding is the only source of fatty acids for the child [17]. At the same time, the same route is used by toxic substances that enter the body of the child, including lead.

Therefore, the aim of this study was to investigate the effect of environmental exposure of mothers to lead, on Pb levels in their milk and blood and in the blood of newborns. Moreover, the aim was to determine the fatty acid profile in the mothers’ blood and milk and in the blood of newborns. We also wanted to find if infant birth weight depends on Pb blood levels, as well as on Pb and fatty acid levels in the blood and milk of the mothers. Finally, we examined if the mothers’ weight and body mass index (BMI) before pregnancy influenced the concentration of Pb and fatty acid profile in the blood and milk of mothers and in the blood of their children.

## Material and Methods

### Characteristics of the Study Group

In order to conduct this study, we obtained the consent of the Bioethics Committee at the Pomeranian Medical University in Szczecin (No. BN-001/02/07). Before giving written consent, each patient received full information about the purpose and plan of study.

The study involved 53 female patients of the Clinic of Obstetrics and Perinatology of the Pomeranian Medical University in Szczecin and their newborns. The patients come from urban areas. The survey was conducted from June to August in 2007 and 2008. Forty-nine newborns were born vaginally and four by caesarean section due to the lack of progress of labor. Each pregnant woman was qualified for the study after exclusion of any pathology in pregnancy and coexisting chronic diseases which preceded the pregnancy. The mean age of patients was 29.11 years. During the study, 32 women were primigravidas, while 21 women were multigravidas. None of the women had had multiple pregnancies, while nine subjects had a history of miscarriages. Fifty newborn babies were born after 37 weeks of gestation, two in the 37th week and one at the 36th week of gestation. All children exhibited functional and morphological maturity, adequate for gestational age (Table 1).

**Table 1:** Descriptive statistics (*n* = 53)

Variable	*x* ± SD	Median	Min-max	CV (%)
Maternal age	29.11 ± 4.77	29.00	18.00–39.00	0.52103
Neonatal birth weight (g)	3511.70 ± 497.75	3570.00	2470.00–4350.00	0.38984
Maternal body weight before pregnancy (kg)	62.94 ± 12.12	61.00	44.00–100.00	0.54997
Maternal prepregnancy BMI	22.60 ± 3.85	21.48	17.63–34.37	0.54997
Postpartum maternal weight (kg)	79.69 ± 13.76	77.00	57.70–120.00	0.49581
Postpartum BMI	28.64 ± 4.48	27.93	21.99–42.52	0.44223

### Collection of Blood and Milk

Blood samples were collected from mothers in the first stage of labor; 9 ml of blood was collected from the cubital vein into tubes containing EDTA as an anticoagulant. Also, 9 ml of blood was collected from umbilical vein into tubes with EDTA after the cord separation. The collected samples were placed at 4 °C and transported within 10 min to the laboratory of the Department of Biochemistry of the Pomeranian Medical University. There they were centrifuged at 1800×*g* for 10 min at 4 °C. The plasma obtained by centrifugation was purged with nitrogen, then frozen and stored at −80 °C until analysis of a given material.

At 5–6 weeks postpartum, an Avent manual breast pump was used to sample approx. 30 mL of breast milk. Milk samples were taken in the morning, after feeding. The samples were then transported on ice to the laboratory of the Department of Biochemistry of the Pomeranian Medical University and purged with nitrogen. After this, the samples were frozen at −80 °C within 1 h of collection until analysis.

### Extraction and Analysis of Fatty Acids

Lipids from the samples were extracted using the Folch mixture. Then, the samples were hydrolyzed to give fatty acids which were transformed into fatty acid methyl esters (FAMEs). By adding 0.5 % solution of BHT, fatty acids were secured against the oxidation reaction. FAME in hexane solution was injected onto the capillary column (CP-SIL88 50 M × 0.25 mm ID, film thickness 0.2 μm, Varian) of the 6890M Agilent gas chromatograph equipped with an autosampler. FAMEs were mobile in the column in an atmosphere of hydrogen as a carrier gas. The initial temperature was about 100 °C and maintained for 1 min, then the temperature was increased at a rate of 10 °C/min to 180 °C, then at a rate of 3 °C/min to 205 °C, and then at a rate of 10 °C/min to 220 °C. Identification of geometric and positional fatty acid isomers was carried out on the basis of a comparison of retention times with the Sigma-Aldrich fatty acid standards. The concentrations of fatty acids were determined based on standard curves and expressed in milligrams per milliliter.

Further analyses took into account the following fatty acids: elaidic (C18:1, 9t), oleic (C18:1, 9c), vaccenic (C18:1, 11t), *cis*-vaccenic (C18:1, 11c), linoleic (C18:2 c), γ-linolenic (C18:3, n-6), α-linolenic (C18:3, n-3), arachidonic (C20:4, n-6), eicosapentaenoic (C20: 5, n-3), and docosahexaenoic acid (C22:6, n-3).

### Determination of the Concentration of Lead in Blood and in Milk

The concentration of lead in whole blood (B-Pb) was measured by means of graphite furnace atomic absorption spectrometry. The procedure was based on the Stoepper et al. method for determination of lead in deproteinized blood samples by electrothermal atomization technique [18]. After deproteinization of blood with 0.8 M nitric acid, the samples were analyzed using the Perkin Elmer 4100ZL atomic absorption spectrometer equipped with Zeeman background correction system and autosampler. The calibration was performed by matrix-match calibration standards. The detection limit was 2 μg/L and precision of the method σ% = 4.4. The results for BCR 194 and BCR 195 certificate reference materials with certificated values of mean 122–130 and 407–425 μg/L were 123 ± 5 and 408 ± 15 μg/L (*n* = 23), respectively. The analysis was performed at the Institute of Occupational Medicine and Environmental Health in a laboratory that regularly participated in two proficiency tests (Lead and Multielement Proficiency—CDC in Atlanta and METOS Program—Istituto Superiore di Sanita in Rome) and fulfilled the requirements of the study organizers.

Lead in milk was determined after mineralization 1 mL of milk with 1 mL of concentrated nitric acid (Baker Instra-Analyzed) under pressure. The samples were subsequently evaporated nearly to dryness and redissolved to 1 mL with 0.8 M nitric acid. The resulted solution was analyzed for lead concentration with graphite furnace atomic absorption spectrometry technique (Perkin Elmer 4100ZL) against aqueous standards prepared on diluted (0.8 M) nitric acid. The detection limit was 0.5 μg/L and trueness based on Seronorm (201405) control material (due to the lack of Certified Reference Materials in liquid form for trace elements) with declared value for lead 2.9 ± 0.2 μg/L was 92 %.

### Statistical Analysis

Anthropometric data and concentrations of selected acids in milk and blood of mothers and children were presented as arithmetic means (*x* ± SD), medians, and in ranges (max-min). Based on body mass and height (B-v) of mothers, BMI was calculated (BMI = body weight [kg] / height of the body [m]^2^) for the time before pregnancy and after childbirth. The results were interpreted based on the WHO classification, where BMI ≤18.49 is insufficient body weight, BMI 18.50–24.99 is a normal range, BMI ≥25.00–29.99 denotes overweight, and BMI ≥30.00 means obesity [19].

The distribution of variables was evaluated using the Shapiro-Wilk *W* test. As the distribution of variables was not deviated from normal distribution, parametric tests were used for further analyses. Pearson correlation analysis was performed to determine the strength of correlation between the variables. The average concentrations in the analyzed milk, maternal blood, and fetal blood were compared by one-way analysis of variance (ANOVA) for variables with a normal distribution. When the analysis of variance showed that the compared means differed significantly, post hoc tests were performed to investigate differences between the means of each group. Comparisons were made with the Tukey’s range test. Tests were evaluated at the significance level *p* ≤ 0.05.

## Results

### Body Mass Index

Before pregnancy, 11.3 % (*n* = 6) of the surveyed women had an insufficient weight, 64.2 % (*n* = 34) had a weight in a normal BMI range, 17 % (*n* = 9) were overweight, and 7.5 % (*n* = 4) were obese, according to BMI classification.

### Pb Concentration in Maternal and Neonatal Blood and in Milk

**Fig. 1:**
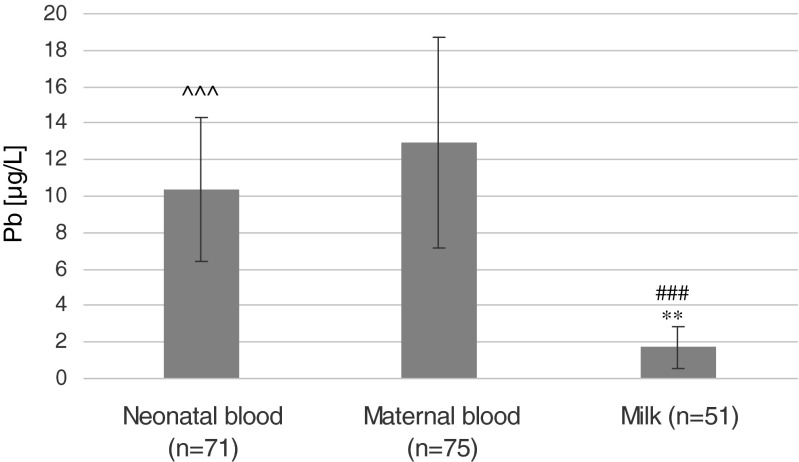
The concentration of lead (Pb) in maternal and neonatal blood and in milk. **Statistically significant difference between the level of Pb in the mother’s blood and breast milk (*p* < 0.001). ###Statistically significant difference between the content of Pb in the neonatal blood and in the milk (*p* < 0.0001). ^^^Statistically significant difference between the content of Pb in the blood of mother and neonate (*p* < 0.0001)

The highest average concentration of Pb was found in maternal blood (1.290 μg/dL ±0.578) and the lowest in milk (0.174 μg/dL ±1.15). On the basis of correlation analysis, we demonstrated a significant and strong correlation between the concentration of maternal blood Pb and neonatal blood Pb (Rs = 0.61; *p* ≤ 0.0001). We also found a weak but statistically significant (Rs = 0.31; *p* = 0.048) correlation between the concentration of Pb in the neonatal blood and the concentration of vaccenic acid (C18: 1, 9t) in maternal blood (Fig. 1).

### Elaidic Acid (C18: 1, 9t) in Maternal and Neonatal Blood and in Milk

The highest average concentration of elaidic acid was determined in milk (0.273 mg/mL ±0.19). The lowest concentration of the acid was observed in the neonatal blood (0.002 mg/mL ±0.001).

Correlation analysis revealed a significant and strong correlation between elaidic and vaccenic acid levels in the mothers’ blood (Rs = 0.85; *p* = 0.0001) and milk (Rs = 0.55; *p* = 0.0001). A weaker correlation between these acids occurred in the neonatal blood (Rs = 0.45; *p* = 0.001). A significant correlation was also found between elaidic acid and γ-linolenic acid in milk (Rs = 0.48; *p* = 0.0001) (Table 2).

**Table 2:** The content of fatty acids in maternal and neonatal blood and in milk

Fatty acid	Neonatal blood (mg/mL)	Maternal blood (mg/mL)	Milk (mg/mL)
Elaidic acid *C18*:*1*, *9t*	0.002 ± 0.001^^^^^	0.014 ± 0.009	0.273 ± 0.194^***,###^
Oleic acid *C18*:*1*, *9c*	0.173 ± 0.054^^^^^	1.065 ± 0.305	15.068 ± 6.913^***,###^
Vaccenic acid *C18*:*1*, *11c*	0.020 ± 0.009^^^^^	0.079 ± 0.030	0.931 ± 0.464^***,###^
Vaccenic acid *C18*:*1*, *11t*	0.0003 ± 0.002^^^^^	0.010 ± 0.006	0.292 ± 0.202^***,###^
Linoleic acid *C18*:*2*, *n-6 c*	0.135 ± 0.041^^^^^	1.165 ± 0.253	5.953 ± 3.012^***,###^
γ-Linolenic acid *C18*:*3*, *n-6 c*	0.044 ± 0.002^^^^^	0.052 ± 0.009	0.095 ± 0.030^***,###^
α-Linolenic acid *C18*:*3*, *n-3 c*	0.004 ± 0.003^^^^^	0.058 ± 0.017	0.855 ± 0.466^***,###^
Arachidonic acid *C20*:*4*, *n-6 c*	0.154 ± 0.043^^^^^	0.267 ± 0.070	0.225 ± 0.111^*,##^
Eicosapentaenoic acid *C20*:*5*, *n-3*	0.005 ± 0.004^^^^^	0.025 ± 0.034	0.039 ± 0.024^***,###^
Docosahexaenoic acid *C22*:*6*, *n-3*	0.058 ± 0.022^^^^^	0.128 ± 0.050	0.127 ± 0.066^###^

^^^Statistically significant difference in the fatty acid level between the maternal and neonatal blood (*p* < 0.0001); ***statistically significant difference in the fatty acid level between the maternal blood and milk (*p* < 0.0001); *statistically significant difference in the fatty acid level between the maternal blood and milk (*p* < 0.01); ###statistically significant difference in the fatty acid level between the neonatal blood and milk (*p* < 0.01); ##statistically significant difference in the fatty acid level between the neonatal blood and milk (*p* < 0.001)

### Oleic Acid (C18: 1, 9c) in Maternal and Neonatal Blood and in Milk

The highest average level of oleic acid was observed in milk (15.068 mg/mL ±6.92), while the smallest in the neonatal blood (0.173 mg/mL ±0.054).

Correlation analysis showed a very strong correlation between oleic acid and C18: 1, 11c in all three cases (milk Rs = 0.95, *p* ≤ 0.01; maternal blood Rs = 0.90, *p* ≤ 0.001; the neonatal blood of Rs = 0.93, *p* ≤ 0.001). A significant (*p* ≤ 0.0001) and also a strong correlation was found between oleic acid and the following acids: linoleic acid in milk (Rs = 0.90), in maternal blood (Rs = 0.73), and neonatal blood (Rs = 0.80); γ-linolenic acid in milk (Rs = 0.83) and in the neonatal blood (Rs = 0.55); α-linolenic acid in milk (Rs = 0.78); arachidonic acid in milk (Rs = 0.91), maternal blood (Rs = 0.63), and neonatal blood (Rs = 0.75); eicosapentaenoic acid in milk (Rs = 0.68); and docosahexaenoic in milk (Rs = 0.71), maternal blood (Rs = 0.62), and neonatal blood (Rs = 0.62). A significant (*p* = 0.0001) and a moderately strong correlation occurred between oleic acid and γ-linolenic acid in the neonatal blood (Rs = 0.55) and α-linolenic acid in maternal blood (Rs = 0.47).

### *Cis*-Vaccenic Acid (C18: 1, 11c) in Maternal and Neonatal Blood and in Milk

Also in the case of *cis-*vaccenic acid, the highest average concentrations are found in milk (0.931 mg/mL ±0.464) and the lowest in the neonatal blood (0.020 mg/mL ±0.009).

On the basis of the correlation analysis, we found showed a significant (*p* = 0.0001) and a strong correlation between the acid C18:1, 11c and the following acids: linoleic acid in milk (Rs = 0.84), maternal blood (Rs = 0.65), and neonatal blood (Rs = 0.74); γ-linolenic acid in milk (Rs = 0.78); α-linolenic acid in milk (Rs = 0.71); arachidonic acid in maternal blood (Rs = 0.61) and neonatal blood (Rs = 0.76); eicosapentaenoic acid in milk (Rs = 0.64); and docosahexaenoic acid in milk (Rs = 0.71). There was a significant (*p* = 0.0001) but moderately strong correlation between vaccenic acid in maternal blood (Rs = 0.47), γ-linolenic acid in the neonatal blood (Rs = 0.47) and docosahexaenoic acid in maternal blood (Rs = 0.59) and neonatal blood (Rs = 0.51).

### Vaccenic Acid (C18: 1, 11t) in Maternal and Neonatal Blood and in Milk

The highest average concentration of vaccenic acid has been identified in milk (0.292 mg/mL ±0.202) and the smallest in the neonatal blood (0.0003 mg/mL ±0.002).

The correlation between the concentration of vaccenic acid concentrations and other acids was placed in U.S. preceding acids.

### Linoleic Acid (C18: 2, *cis*) in Maternal and Neonatal Blood and Milk

As in all previous acids, also in the case of linoleic acid, the highest average concentration is found in milk (5.953 mg/mL ±3.012) and the lowest in the neonatal blood (0.135 mg/mL ±0.41).

Correlation analysis revealed a significant (*p* = 0.0001) and strong correlation between linoleic acid and the following acids: γ-linolenic acid in milk (Rs = 0.76); α-linolenic acid in milk (Rs = 0.80) and maternal blood (Rs = 0.66); arachidonic acid in milk (Rs = 0.86), maternal blood (Rs = 0.64), and neonatal blood (Rs = 0.71); eicosapentaenoic acid in milk (Rs = 0.60); and docosahexaenoic acid in the neonatal blood (Rs = 0.69). A significant (*p* = 0.0001) but weaker correlation was found between linoleic acid and γ-linolenic acid in the neonatal blood (Rs = 0.47), α-linolenic acid in neonatal blood (Rs = 0.55), and docosahexaenoic acid in maternal blood (Rs = 0.56).

### γ-Linolenic Acid (C18: 3, n-6 Gamma) in Maternal and Neonatal Blood and in Milk

The greatest concentration of γ-linolenic acid was determined in milk (0.095 mg/mL ±0.030), and the lowest in the neonatal blood (0.044 mg/mL ±0.002).

Based on the analysis of the correlation, a significant (*p* = 0.0001) and strong correlation was observed between γ-linolenic acid and arachidonic acid in milk (Rs = 0.78) and between γ-linolenic acid and eicosapentaenoic acid in milk (Rs = 0.60). A significant (*p* = 0.0001) but statistically less strong correlation occurred between γ-linolenic acid and α-linolenic acid in milk (Rs = 0.54), arachidonic acid in the maternal blood (Rs = 0.51), and docosahexaenoic acid in milk (Rs = 0.49).

### α-Linolenic Acid (C18: 3, n-3 Linolenic Acid) in Maternal and Neonatal Blood and in Milk

The highest average level of α-linolenic acid was determined in milk (0.855 mg/mL ±0.466) while the lowest in neonatal blood (0.004 mg/mL ±0.003).

Correlation analysis revealed a significant (*p* = 0.0001) and a strong correlation between the α-linolenic acid and arachidonic acid in milk (Rs = 0.71), eicosapentaenoic acid in milk (Rs = 0.67), and docosahexaenoic acid also in milk (Rs = 0.63). A significant (*p* = 0.0001) but weaker correlation was found between α-linolenic acid and docosahexaenoic acid in maternal blood (Rs = 0.47) and neonatal blood (Rs = 0.55) (Table 2).

### Arachidonic Acid (C20: 4, n-6) in Maternal and Neonatal Blood and in Milk

Unlike previous acids, the highest average concentration of arachidonic acid was fond in maternal blood (0.267 mg/mL ±0.070), while the lowest in neonatal blood (0.154 mg/mL ±0.043).

Correlation analysis showed a significant (*p* = 0.0001) and strong correlation between the arachidonic acid and eicosapentaenoic acid in milk (Rs = 0.71) and docosahexaenoic acid in milk (Rs = 0.77) and maternal blood (Rs = 0.75). An equally significant (*p* = 0.0001) but weaker correlation was determined between the arachidonic acid and docosahexaenoic acid in neonatal blood of the child (Rs = 0.58) (Table 2).

### Eicosapentaenoic Acid (C20: 5 n-3) in Maternal and Neonatal Blood and in Milk

In the case of eicosapentaenoic acid, the highest average concentration was observed in milk (0.039 mg/mL ±0.024), while the lowest in neonatal blood (0.005 mg/mL ±0.004).

Correlation analysis showed a significant and strong correlation between eicosapentaenoic acid and docosahexaenoic acid in milk (*p* = 0.0001, Rs = 0.78) (Table 2).

### Docosahexaenoic Acid (C22: 6, n-3) in Maternal and Neonatal Blood and in Milk

The highest concentration of docosahexaenoic acid was found in maternal blood (0.128 mg/mL ±0.050). The lowest concentration of the acid occurred in neonatal blood (0.058 mg/mL ±0.022). There were no significant correlations between the studied parameters for docosahexaenoic acid (Table 2).

## Discussion

This is the first study on the effect of environmental exposure to Pb on the fatty acid profile in the mothers’ blood and milk and in the blood of newborns. Our results showed, that Pb-B values in the mothers blood was below levels considered safe for pregnant women, e.g. below 5 μg/dL [3]. Pb content in all blood samples from the newborns were also inside the safe levels. This indicates that mothers were not exposed to high Pb concentrations before and during pregnancy. They were representative for the area characterized by moderate Pb levels in the environment. The results are consistent with previous studies on the transport of Pb through the placenta to the cord blood [20, 21]. Our study also showed a significant and strong correlation between Pb levels in the blood of the mother and the child. This supports the idea that the transport of Pb through the placenta is neither regulated nor selective [21]. A woman’s body during pregnancy is burdened with additional demands. It must create the right conditions for the growth of the fetus and provide it with all the elements necessary for development—hence the importance of a correct diet. One of the nutrients necessary for the proper development of the fetus is lipids. The lipid profile of a pregnant woman changes to ensure energy security for both her and the fetus. The mother’s body increases its absorption of lipids and raises their concentration in the blood. The concentrations of total cholesterol and VLDL, LDL, HDL, and TG increase along with the development of gestation [16].

In the body of the developing fetus, fatty acids are transferred mainly to the cytoplasm of fat cells, where they can be subjected to oxidation, multiplication of double bonds in the molecule, or extension of the molecule. The other main site of fatty acid occurrence is cellular structures, especially phospholipids. The type of acid that is built in phospholipids decides the fluidity of the membrane structure and function of membrane receptors and enzymes. Studies have shown that dietary *trans*-unsaturated fatty acids (TFAs) are more actively incorporated into the cell membrane than *cis*-unsaturated fatty acids [17]. Although placental transfer of saturated and *cis*-unsaturated fatty acids has been demonstrated in rats, very few studies have investigated the placental transport of *trans*-fatty acids, and the obtained results are contradictory [22–24].

A study of Larqué et al. [25] shows that *trans*-fatty acids are incorporated into plasma and liver microsomes of pregnant rats in high concentrations and according to their profile and content in the diets; however, this is shown not to occur in the brain. The placenta also incorporates high amounts of *trans*-isomers into its structure. However, this barrier is not completely impermeable, inasmuch as a number of *trans*-fatty acids cross the barrier and accumulate in the liver of the fetus, showing a clear exposure of fetal tissues to maternal dietary *trans*-fatty acids. This reflects the protective mechanisms serving to limit the incorporation of TFAs in the central nervous system [25, 26].

However, it is uncertain whether exposure to TFA in early human life has adverse consequences. Studies on animals show different explanations of TFA incorporation in human tissues and the biological consequences of this process in the neonatal period and during lactation. It has been shown that animals born by mothers fed a diet rich in TFA had significantly lower concentrations of those acids in their blood compared to maternal blood. Some amounts of *trans*-FAs reach the placenta but are probably catabolized in fetal tissues or replaced by the newly synthesized FAs [26]. In our research, we also observed a much lower concentration of FAs in the children’s blood compared to maternal blood. This may be a confirmation of the proposition that FA are metabolized in placenta tissues or that there are some mechanisms inhibiting the transport of high FA concentrations through the placenta. In our study, however, we found no correlation between individual fatty acids in the mother’s blood and baby’s blood.

The concentration of fats in milk increases significantly during breast-feeding. The main fatty acids in milk are saturated fatty acids, half of which is palmitic acid. These acids can be synthesized de novo in the tissues of the body or may be provided with the diet of mother. *Cis*-unsaturated fatty acids are mainly represented by 18:1 9c (oleic acid). Although monoene fatty acids are not essential and can be synthesized, their intake with diet may have positive physiological effects on the fluidity of cell membranes and cholesterol metabolism.

The content of *trans*-unsaturated fatty acids in human breast milk is estimated to be 4.4 %, with the main *trans*-isomer being C18:1 11t (vaccenic acid), followed by isomers 14:1t, 16:1t, and 20:1t [27]. The concentrations of unsaturated fatty acids in our study showed the highest concentration of oleic acid and the smallest of eicosapentaenoic acid (Table 2). Vaccenic and elaidic acids had the highest concentrations among *trans*-unsaturated fatty acids.

The types of lipids present in breast milk depend on the diet [17, 28], similar to the total amount of TFA. Their presence may limit the availability of long-chain polyunsaturated fatty acids (LC-PUFAs). Because LC-PUFAs are very important for the early development of eyesight and cognitive abilities, factors that reduce their availability after birth are a serious issue [29]. However, until recently, this role of LC-PUFAs has been controversial. Some studies show an inverse correlation between the concentration of TFA 18:1 and PUFAs n-3 and n-6 in breast milk and blood of the newborn. Desci et al. [28] showed that in the lipids of umbilical vascular walls an increased concentration of TFA C18:1 is associated with decreased levels of docosahexaenoic acid. This relationship has been confirmed by other authors, for example Elias and Innis [30]. However, other studies show no connection between these two groups of fatty acids, similar to the results of the present study [31]. A negative correlation between TFAs and PUFAs results from the inhibition of LC-PUFA synthesis by TFA, which is confirmed in research conducted on rats and in vitro on rat tissues and human fibroblasts. However, in that case, it was impossible to make a clear statement because of other works which show that the inhibitory action of TFAs is observed only at high levels which are not normally found in the diet [17].

The inhibitory effect is postulated to be associated with disorders of enzymatic conversion of linoleic acid to arachidonic acid, documented in studies on animal models and in human fibroblasts in vitro. It is assumed that TFAs are incorporated into the cell membrane and inhibit the metabolic conversion of 18:2n-6 and 18:3n-3 to the longer chains of n-6 and n-3 PUFAs. A significant decrease of n-6 and n-3 acids observed in this study represents the effect of TFAs on the desaturation and chain elongation of 18:2n-6 and 18:3n-3, which indicates a loss of metabolic properties [32]. The current study demonstrates that there is an inverse correlation not only between TFAs and LC-PUFAs as well as between TFAs and the substrate/product ratio in the synthesis of LC-PUFAs but also between TFAs and linoleic acid in cholesterol esters. However, it is not known yet whether this could have a connection with the inhibition of the biosynthesis or if the result is a combination of these two processes [29].

One of the dependencies that we investigated in this study was the correlation between the studied FAs and BMI before and after pregnancy. The results of our study showed no such correlation. FAs did not correlate with maternal body weight before and after pregnancy. This may have been due to the fact that the BMI of most of the surveyed women did not indicate obesity and were within the normal range BMI = 18.5–24.99. Other publications, however, show a correlation between BMI before pregnancy and mother’s lipid profile during the initial phase of gestation, which also correlated with the child’s obesity [33].

Also controversial is the question of the effects of inhibition of the synthesis of linoleic acid and α-linolenic acid by TFAs. It is assumed that it may result in defects in fetal development and low birth weight. Some authors claim that inhibiting the synthesis of the aforementioned acids results in a shorter pregnancy, having no effect on birth weight and length of the body of the newborn. Others believe that the result is lower birth weight and length of the body with a normal duration of pregnancy.

A third position assumes that these three values are reduced, i.e. that the effects include low birth weight, shorter body length, and a shorter duration of pregnancy. On this basis, it is difficult to determine what exactly is affected by TFA-induced inhibition of the synthesis of linoleic acid and alpha-linolenic acid, although a negative impact on fetal development is visible [17]. The results of our study showed no correlation between the studied FAs and birth weight.

In this study, no correlation was observed between Pb blood levels and the tested FAs, except for one case of a weak correlation between the Pb in the blood of the newborn and elaidic acid concentration in maternal blood. It is difficult to discuss the obtained results because there are no studies on the relationship between the concentration of blood FAs and Pb in available literature. There are only reports on the link between psychosocial disorders of children and the concentrations of Pb and FAs, which indicate a possible relationship between these two variables. The authors of that paper showed that children with elevated levels of blood Pb exhibited disorders in psychosocial functioning at school and at home. The same behavior was revealed in children with a reduced concentration of arachidonic acid, γ-linolenic acid, eicosapentaenoic acid (EPA), and docosahexaenoic acid (DHA) levels [33]. To find a definite link between psychosocial disorders of children and the concentrations of Pb and FAs, further research is required to study correlations of Pb and TFA and the mechanisms of their actions in the human body.

In our study, we found no correlation between maternal blood Pb and low birth weight. This was probably due to the low concentration of Pb in the blood of mothers, insufficient to induce any such effect.

Exposure of children, adolescents, and adults to Pb negatively correlates with BMI and obesity [34, 35]. Also our study confirmed this correlation between the concentration of Pb in the blood of mothers and mothers’ BMI before pregnancy.

## Conclusion

Our study established a significant and strong correlation between the content of Pb in the blood of the mother and the child. This supports the assumption that the transport of Pb through the placenta is neither regulated nor selective. Environmental maternal exposure to lead resulting in Pb-B within levels previously considered safe for pregnant women, i.e., 5 μg/dL, had no effect on the concentration of fatty acids in the blood and milk of mothers or in the blood of newborns.

## References

[1] CDC 2004 (Center for Disease control and Prevention). National Center for Environmental Health. A review of evidence of health effects of blood lead levels <10 μg/dL in children. Atlanta. http://www.cdc.gov/nceh/lead/ACCLPP/meetingMinutes/lessThan10MtgMAR04.pdf Access 26 March 2015

[2] CDC (Centers for Disease Control and Prevention) (2005) National Center for Environmental Health. Preventing lead poisoning in young children. Atlanta

[3] CDC (Centers for Disease Control and Prevention) (2012) Sources of lead. Available: http://www.cdc.gov/nceh/lead/tips/sources.html Access 26 March 2015; EP (Environmental Protection) (2005) The restriction of the use of certain hazardous substances in electrical and electronic equipment regulations No. 2748. http://www.legislation.gov.uk/uksi/2005/2748/pdfs/uksi_20052748_en.pdf Access 26 March 2015

[4] WHO (World Health Organization), 2010. Preventing disease through healthy environments. Available: http://www.who.int/ipcs/features/10chemicals_en.pdf Access 26 March 2015; EP (Environmental Protection) (2009) The restriction of the use of certain hazardous substances in electrical and electronic equipment (amendment) regulations, No. 581. http://www.legislation.gov.uk/uksi/2009/581/pdfs/uksi_20090581_en.pdf Access 26 March 2015

[5] EU. 2008 (European Commission). Institute for Health and Consumer Protection Toxicology and Chemical Substances (& ECB). Opinion of the TC NES on the Environment Part of Industry Voluntary Risk Assessments on Lead and Lead Compounds. http://echa.europa.eu/doc/trd_substances/VRAR/Lead/tcnes_opinion/tcnes_opinion_env.pdf Access 26 March 2015

[6] CDC (Centers for Disease Control and Prevention), 2002. Managing elevated blood lead levels among young children: recommendations from the Advisory Committee on Childhood Lead Poisoning Prevention. Atlanta, GA:U.S. Department of Health and Human Services, CDC. Available: http://www.cdc.gov/nceh/lead/casemanagement/casemanage_main.htm Access 26 March 2015

[7] Moszyński P (2010). Lead poisoning in Nigeria causes “unprecedented” emergency. BMJ.

[8] Lo YC, Dooyema CA, Neri A, Durant J, Jefferies T, Medina-Marino A, de Ravello L, Thoroughman D, Davis L, Dankoli RS, Samson MY, Ibrahim LM, Okechukwu O, Umar-Tsafe NT, Dama AH, Brown MJ (2012). Childhood lead poisoning associated with gold ore processing: a village-level investigation-Zamfara State, Nigeria, October-November 2010. Environ Health Perspect.

[9] PubChem Public Chemical Database; http://pubchem.ncbi.nlm.nih.gov/compound/5352425?from=summary#section=Toxicity, Access 26 March 2015

[10] WHO, http://www.who.int/ipcs/features/lead.pdf Access 18 November 2013

[11] Chelchowska M, Ambroszkiewicz J, Jablonka-Salach K, Gajewska J, Maciejewski TM, Bulska E, Laskowska-Klita T, Leibschang J (2013). Tobacco smoke exposure during pregnancy increases maternal blood lead levels affecting neonate birth weight. Biol Trace Elem Res.

[12] WHO. Environmental Health Criteria 165. Inorganic lead. Geneva. 1995

[13] Afeiche M, Peterson KE, Sánchez BN, Schnaas L, Cantonwine D, Ettinger AS, Solano-González M, Hernández-Avila M, Hu H, Téllez-Rojo MM (2012). Windows of lead exposure sensitivity, attained height, and body mass index at 48 months. J Pediatr.

[14] Baranowska-Bosiacka I, Strużyńska L, Gutowska I, Machalińska A, Kolasa A, Kłos P, Czapski GA, Kurzawski M, Prokopowicz A, Marchlewicz M, Safranow K, Machaliński B, Wiszniewska B, Chlubek D (2013). Perinatal exposure to lead induces morphological, ultrastructural and molecular alterations in the hippocampus. Toxicology.

[15] CDC (Centers for Disease Control and Prevention), 2007. Interpreting and managing blood lead levels <10 μg/dL in children and reducing childhood exposures to lead: recommendations of CDC’s Advisory Committee on Childhood Lead Poisoning Prevention. MMWR Recomm Rep 56, 1–16. Available: http://www.cdc.gov/mmwr/preview/mmwrhtml/rr5608a1.htm Access 26 March 2015 17975528

[16] Lippi G, Albiero A, Montagnana M, Salvagno GL, Scevarolli S, Franchi M, Guidi GC (2007). Lipid and lipoprotein profile in physiological pregnancy. Clin Lab.

[17] Jamioł-Milc D, Stachowska E, Chlubek D (2010). The effects of dietary *trans* fatty acids in pregnancy and lactation. Annales AMS.

[18] Stoeppler M, Brandt K, Rains T.C. (1978). Contribution to automated trace analysis. Part II. Rapid method for the automated determination of lead in whole blood by electrothermal atomic-absorption spectrophotometry. Analyst.

[19] Physical status (1995) The use and interpretation of anthropometry. Report of a WHO expert committee. WHO Tech Rep Ser Geneva 854:1–452 8594834

[20] Krzywy I, Krzywy E, Pastuszak-Gabinowska M, Brodkiewicz A (2010). Lead—is there something to be afraid of?. Annales AMS.

[21] Suprewicz K, Kozikowska I, Chrobaczyńska-Dyląg M, Gał A, Piekarz A, Sikora J, Sławska H, Stawarz R (2013). Effects of the cigarette smoking on the newborn clinikalclinical parametrsparameters and the accumulation of cadmium and lead in the placenta of women from Upper Silesia. Ginekol Pol.

[22] Johnston PV, Kummerow FA, Walton GH (1985). Origin of the trans fatty acids in human tissue. Proc Soc Exp Biol Med.

[23] Senti FR, Senti FR (1985). Health aspects of dietary trans fatty acids. Life Sciences Research Office.

[24] Koletzko B, Muller J (1990). Cis- and trans-isomeric fatty acids in plasma lipids of newborn infants and their mothers. Biol Neonate.

[25] Larque E, Pérez-Llamas F, Puerta V, Girón MD, Suárez MD, Zamora S, Gil A (2000). Dietary trans fatty acids affect docosahexaenoic acid concentrations in plasma and liver but not brain of pregnant and fetal rats. Pediatr Res.

[26] Larque E, Zamora S, Gil A (2001). Dietary trans fatty acids in early life: a review. Early Hum Dev.

[27] Koletzko B, Mrotzek M, Bremer HJ (1988). Fatty acid composition of mature human milk in Germany. Am J Nutr.

[28] Berghaus TM, Demmelmair H, Koletzko B (1998). Fatty acid composition of lipid classes in maternal and cord plasma at birth. Eur J Pediatr.

[29] Decsi T, Burus I, Molnár S, Minda H, Veitl V (2001). Inverse association between trans isomeric and long-chain polyunsaturated fatty acids in cord blood lipids of full-term infants. Am J Clin Nutr.

[30] Elias SL, Innis SM (2001). Infant plasma trans, n-6, and n-3 fatty acids and conjugated linoleic acids are related to maternal plasma fatty acids, length of gestation, and birth weight and length. Am J Clin Nutr.

[31] Mahomed K, Williams M, King I, Mudzamiri S, Woelk G (2007). Erythrocyte omega-3, omega-6 and trans fatty acids in relation to risk of preeclampsia among women delivering at Harare Maternity Hospital, Zimbabwe. Physiol Res.

[32] Kummerow FA, Zhou Q, Mahfouz MM, Smiricky MR, Grieshop CM, Schaeffer DJ (2004). Trans fatty acids in hydrogenated fat inhibited the synthesis of the polyunsaturated fatty acids in the phospholipid of arterial cells. Life Sci.

[33] Gademan M, Vermeulen M, Oostvogels A, Roseboom TJ, Visscher T, van Eijsden M, Twickler MT, Vrijkotte TG. (2014) Maternal prepregnancy BMI and lipid profile during early pregnancy are independently associated with offspring’s body composition at age 5–6 years: the ABCD study. PLoS One 9(4):e94594 10.1371/journal.pone.0094594 PMC3989215 24740157

[34] Scinicariello F, Buser MC, Mevissen M, Portier CJ (2013) Blood lead level association with lower body weight in NHANES 1999-2006. Toxicol Appl Pharmacol 273(3):516–523 10.1016/j.taap.2013.09.022 PMC8158151 24099784

[35] Jain RB (2013). Effect of pregnancy on the levels of blood cadmium, lead and mercury for females aged 17-39 years old. J Toxicol Environ Health A.

